# Predictors of multidisciplinary rehabilitation outcomes in patients with chronic musculoskeletal pain: protocol for a systematic review and meta-analysis

**DOI:** 10.1186/s13643-017-0598-0

**Published:** 2017-10-11

**Authors:** Elena Tseli, Wilhelmus Johannes Andreas Grooten, Britt-Marie Stålnacke, Katja Boersma, Paul Enthoven, Björn Gerdle, Björn Olov Äng

**Affiliations:** 1 Department of Neurobiology, Care Sciences and Society, Division of Physiotherapy, Karolinska Institutet, 23100, 141 83 Huddinge, Sweden; 2 Functional Area Occupational Therapy and Physiotherapy, Allied Health Professionals Function, Karolinska University Hospital, Stockholm, Sweden; 3 Department of Community Medicine and Rehabilitation, Rehabilitation Medicine, Umeå University, Umeå, Sweden; 4 Department of Clinical Sciences, Department of Rehabilitation Medicine, Karolinska Institutet, Danderyd Hospital, Stockholm, Sweden; 5 School of Law, Psychology and Social Work, Örebro University, Örebro, Sweden; 6 Department of Medical and Health Sciences, Linköping University, Linköping, Sweden; 7 Pain and Rehabilitation Centre, Department of Medical and Health Sciences, Linköping University, Linköping, Sweden; 8 School of Education, Health and Social Studies, Dalarna University, Falun, Sweden

**Keywords:** Chronic musculoskeletal pain, Literature review, Meta-analysis, Multidisciplinary rehabilitation, Prognostic factors, Treatment outcome

## Abstract

**Background:**

Chronic musculoskeletal pain is a major public health problem. Early prediction for optimal treatment results has received growing attention, but there is presently a lack of evidence regarding what information such proactive management should be based on. This study protocol, therefore, presents our planned systematic review and meta-analysis on important predictive factors for health and work-related outcomes following multidisciplinary rehabilitation (MDR) in patients with chronic musculoskeletal pain.

**Methods:**

We aim to perform a synthesis of the available evidence together with a meta-analysis of published peer-reviewed original research that includes predictive factors preceding MDR. Included are prospective studies of adults with benign, chronic (> 3 months) musculoskeletal pain diagnoses who have taken part in MDR. In the studies, associations between personal and rehabilitation-based factors and the outcomes of interest are reported. Outcome domains are pain, physical functioning including health-related quality of life, and work ability with follow-ups of 6 months or more. We will use a broad, explorative approach to any presented predictive factors (*demographic*, *symptoms-related*, *physical*, *psychosocial*, *work-related, and MDR-related*) and these will be analyzed through (a) narrative synthesis for each outcome domain and (b) if sufficient studies are available, a quantitative synthesis in which variance-weighted pooled proportions will be computed using a random effects model for each outcome domain. The strength of the evidence will be evaluated using the Grading of Recommendations, Assessment, Development and Evaluation.

**Discussion:**

The strength of this systematic review is that it aims for a meta-analysis of prospective cohort or randomized controlled studies by performing an extensive search of multiple databases, using an explorative study approach to predictive factors, rather than building on single predictor impact on the outcome or on predefined hypotheses. In this way, an overview of factors central to MDR outcome can be made and will help strengthen the evidence base and inform a wide readership including health care practitioners and policymakers.

**Systematic review registration:**

PROSPERO CRD42016025339

**Electronic supplementary material:**

The online version of this article (10.1186/s13643-017-0598-0) contains supplementary material, which is available to authorized users.

## Background

Chronic musculoskeletal pain (i.e., pain duration > 3 months) such as chronic neck/shoulder and back pain, or generalized widespread pain, is a major health and socioeconomic burden. About a quarter of the global adult population live with chronic pain of significant intensity [1], which results in poor health including psychological distress, reduced quality of life, impaired physical functioning, reduced work ability, and increased sick leave [2]. Years lived with disability (YLD) is a measure of non-fatal health outcomes, and pain conditions cause 21% of all YLDs globally. In a large meta-analysis of the global burden of disease published in 2015, low back pain emerged as the leading single cause of YLD, followed by major depression disorders, anemia, and neck pain [3]. In addition, societal costs of chronic pain are immense. In Sweden, direct and indirect costs have been estimated to be €9.6 billion annually by the Swedish Council on Technology Assessment [4]. Despite this, few policies have aimed specifically at chronic pain as a public health problem, which has been noted in recent literature [1].

From a therapeutic perspective, chronic musculoskeletal pain is indeed a complex, multifaceted condition. A biopsychosocial approach is necessary for understanding and treating chronic pain—as a result, multidisciplinary rehabilitation (MDR, also known as multimodal rehabilitation), a comprehensive interdisciplinary pain management method, is advised for this patient group. Based on a cognitive-behavioral therapy approach, it incorporates education, physical activity/exercise, coping skills, and occupational therapy sessions. It is administered by multidisciplinary teams that commonly include physicians, psychologists, physiotherapists, occupational therapists, social workers, and other health professionals [5]. Its individual treatment modules are expected to function both independently and in conjunction with other modules, and the effects are intended to be greater than the sum of its components. It corresponds to what the Medical Research Council guidance has defined as a complex intervention [6]. These biopsychosocial rehabilitation programmes are consequently intended to target most facets of the chronic pain condition, which is why multiple outcomes should be considered when evaluating the efficacy and effectiveness of such treatments.

With the development of a core set of outcome domains, the Initiative on Methods, Measurement, and Pain Assessment in Clinical Trials (IMMPACT) highlighted six important and valued topics for evaluation in chronic pain clinical trials—to facilitate comparison across studies: pain, physical functioning, emotional functioning, participant ratings of improvement and satisfaction with treatment, adverse events, and participant disposition [7]. In addition to pain reduction, broad aspects of both physical and emotional functioning are thus emphasized, including health-related quality of life and work ability as subsets to physical functioning [8]. Applying the perspective of domains in the International Classification of Functioning, Disability, and Health (ICF), these aspects could also be viewed in a “body function–activity–participation” context [9], to further add to the complexity of evaluating outcomes of MDR.

Providing evidence and predictions for MDR in patients with chronic pain has been recognized as a major challenge due to the complexity of the conditions and diverse nature of the MDR components. To date, there is little scientific literature on how MDR interventions should be designed to optimize results [4]. It is still unknown what treatment components are really important, and whether all components benefit patients alike [4, 10]. This lack of evidence is causing uncertainty, which delays the improvement of existing MDR programmes. The need for more knowledge in the area has received attention since MDR is expensive and time-consuming, and clinical departments actively select which patients are offered this intervention.

It is believed that evidence of relevant and essential predictors (also known as prognostic factors) can provide an important element for further study in clinical trials, and will enable MDR providers to select and customize treatment according to the patient’s profile, thus maximizing outcomes in relation to costs. The gold standard for high-ranking evidence synthesis is systematic literature reviews and meta-analyses, in which aggregated data from individual studies may add to statistical power. However, for our population of interest, i.e., patients with chronic musculoskeletal pain conditions, previous reviews on predictors following MDR in patients with fibromyalgia [11] and low back pain [12, 13] have not permitted pooling of data for meta-analysis, since the included studies were considered to be heterogeneous. In those reviews, the latest including published data until 2010, relatively few studies contributed to summarizing the predictor strength and its potential relationship with a particular outcome. Still, using a qualitative data-synthesis, some predictors were identified, for example, patients’ initial level of depression [11] and pain intensity [11, 12], work-related functioning, and active coping skills at baseline [12]—some of which point in opposite directions.

Adding to the patient-related factors, some factors may relate to the treatment itself, e.g., specific MDR orientation, MDR dose, treatment processes, and interactions between different patient characteristics. Due to the limited number of studies investigating such variations [11, 13], the effects of these still remain unclear, delaying evidence on whether some treatment modalities work better in some types of patients. For this heterogeneous patient population, more adequately tailored rehabilitation programmes could improve rehabilitation success for identified subgroups of high risk that are called for by the health care community [4, 10].

Although etiology, localization, and diagnoses might differ, chronic pain itself could be considered a disease in its own right [14]. Evidence of some generic prognostic factors for musculoskeletal conditions prevalent in primary care has been presented, identifying high pain severity, multisite pain, baseline disability, longer pain duration, and older age as potential prognostic factors for disability across pain sites [15, 16]. However, there is limited confidence in the results. Furthermore, when a range of biopsychosocial and sociodemographic variables was investigated in pain populations with more mixed chronicity [17] in chronic samples, once again, the limited number of studies did not allow the authors to obtain strong evidence for any prognostic factor.

Hence, clinicians still lack evidence on whether specific patient characteristics, with worse or better baseline status, are of any significance for treatment outcomes of MDR for informed decisions with regard to the best timing, content, or duration of the intervention.

A thorough and systematic overview of all studied factors that might predict important outcomes following MDR interventions is therefore needed. Consequently, to increase power, this should take into consideration chronic musculoskeletal pain diseases as a group. Such an overview will, hopefully, add knowledge on how MDR may be tailored to patients’ resources and limitations. Providing MDR pain centers with tools and knowledge to optimize the fit between appropriate treatment content, intensity, and patient needs will reduce unnecessary costs arising from, for example, overdosing or generalizing treatment. Simultaneously, if patient needs are met more accurately, higher satisfaction with the treatment and improved treatment outcomes should result.

### Objectives

Our overall objective is to identify, evaluate, and meta-synthesize published data on predictive factors for positive outcomes in patients with chronic musculoskeletal pain following multidisciplinary rehabilitation.

## Methods

The present study protocol describes an ongoing systematic review and meta-analysis of prognostic factors on clinical outcomes following MDR. The protocol adheres to the requirements of Preferred Reporting Items for Systematic Reviews and Meta-Analyses protocols (PRISMA-P) [18], with a populated PRISMA-P 2015 checklist included as an additional file [see Additional file 1], in the interest of transparency and completeness. The review will conform to the related PRISMA guidelines [19]. We registered this systematic review in PROSPERO, the International Prospective Register of Systematic Reviews, on February 5, 2016 (ref id: CRD42016025339) and the project is ongoing (May 31, 2017).

### Eligibility criteria

Included in this review are articles on longitudinal studies that report empirical data, either observational (cohort, case-control) or experimental/clinical trials (RCT), in which predictive factors are presented from baseline to follow-up. Articles need to be original research papers published in full-text and in peer-reviewed journals, thus studies that reflect commentaries or editorials are excluded together with articles written in languages other than English. We use an explorative approach, aiming to reach all investigated factors potentially predictive of treatment outcome. Below, eligibility criteria are defined according to population, intervention, or variable of interest (as in exposure or predictive factor), comparators/referents, outcomes (PICO).

### Population of interest

Adults aged 18–67 years (i.e., the working-age population) with chronic musculoskeletal pain, who have taken part in multidisciplinary rehabilitation programmes following the biopsychosocial model; defining chronic as a duration of > 3 months and delimitating musculoskeletal pain conditions to those not emanating from malignancies or systemic diseases (e.g., rheumatoid arthritis). The population thus consists of a wide range of common benign chronic pain diagnoses, i.e., patients with back pain, neck pain, and generalized pain syndromes (including fibromyalgia and general widespread pain).

### Variable of interest (as in exposure or predictive factor)

Any independent variable investigated for potential predictive ability. We aim to evaluate all personal, work- and rehabilitation-based factors described in the scientific literature. Our broad study approach will consequently cover a variety of investigated prognostic factors, which will later be grouped into relevant domains. Predictive factors may be “personal”, e.g., demographic factors (sex, age, socioeconomic status, lifestyle), symptoms-related factors (pain intensity, pain duration, comorbidity), physical functioning (self-rated, assessed), psychosocial factors (cognitive, emotional, or social), and work-related factors or “treatment-related” (MDR duration, intensity, or content). A variable of interest might, for example, be the association of high baseline depression level with treatment outcome.

### Comparators

A comparator is the alternative exposure within the predictive factor. The comparators (referents) are, thus, those not exposed to the predictor of interest, for example, those with a low depression level (vs. high depression level) at baseline.

### Outcome

Longitudinal follow-up according to what is recommended by IMMPACT as core outcome measures in subjects with chronic pain [20]. For the purpose of this review, we will primarily focus on pain and physical functioning including measures of health-related quality of life and work ability. Any additional outcomes falling within the IMMPACT recommended outcome domains (emotional functioning, participant ratings of global improvement, and satisfaction with treatment) that are found during the review process will be analyzed elsewhere. Only long-term follow-up data (6 months or more) will be included in the analyses.

### Study identification

The search strategy was developed with the support of a research librarian at Karolinska Institutet University Library, to optimize structure and completeness, covering all necessary descriptors to the topic definition [21, 22]. The search strategy adheres to the aforementioned PICO descriptors, but for purposeful recall, the search parameters were modified to appropriately define the intervention of interest and to filter for studies including prediction analyses. Thus, four search parameters (domains) were set: “chronic pain”–“multidisciplinary rehabilitation”–“treatment outcome”–“prediction”, joined with the Boolean operator “AND”. For each parameter, proper and exhaustive terms were used, identified through the screening of search strategies from previous systematic reviews on similar topics [10–13, 23, 24], from Medical Subject Heading-indexations (MeSH) and search terms of known, relevant primary studies, complemented with browsing of the thesauri of selected databases for additional controlled vocabulary. Validation procedures of sensitivity and specificity for each search parameter were performed to ensure comprehensiveness and relevance of the search strategy [25–27], which was subsequently adapted to every other reference database and peer-reviewed by the research librarian in the final stage.

### Information sources

The six electronic databases MEDLINE and PsycINFO (via Ovid), EMBASE (via Elsevier), CINAHL (via EBSCO), Web of Science (via Thomson Reuters), and the Cochrane Central Register of Controlled Trials (CENTRAL) were searched in September 2015 to identify studies published from 1980 until that date. The search was later updated to include additional studies published up to April 2017. To maximize recall, the search was unrestricted except for the two limitations publication language and publication time. Our search strategy for MEDLINE (Ovid) is presented in an additional file [see Additional file 2]. The sample search strategy was translated into database-specific syntax for the other databases used. The reference lists of included studies and of related review papers will also be examined for additional records.

### Study selection

An interdisciplinary review team, consisting of six members with expertise in multidisciplinary rehabilitation and chronic pain, was compiled for the selection process. The process of decision-making for inclusion based on the eligibility criteria was first piloted on a small sample of articles to validate the criteria and interpretation of studies.

The selection process will be performed in four steps:

1. *Screening of titles*: removal of clearly unrelated records, one reviewer (ET).
2. *Screening of abstracts*: independent assessment by two reviewers (BMS and KB), any conflicts to be resolved by a third reviewer (ET).
3. *Screening of full texts for PICO eligibility*: two pairs of reviewers (BMS and ET, KB and PE) will each screen half of the articles. Each article will be independently appraised by the two reviewers it is assigned to, and disagreements will be resolved through discussions with the full review team.
4. *Screening of full texts for Relevance according to study objective*: all remaining articles will be assessed once more by three senior reviewers (BMS, KB, BG), for fulfillment of relevance criteria according to an objective—compliant protocol, which has been developed by the authors (available from the corresponding author on request). One senior reviewer (BG) will examine all studies, while the other two (BMS and KB) will re-examine half of the studies each. To date, we have finalized the selection procedure from the first database search (Sept 2015) and have proceeded to step 3, screening of full texts, with the additional records retrieved from the second database search (April 2017).

### Data management

**Fig. 1:**
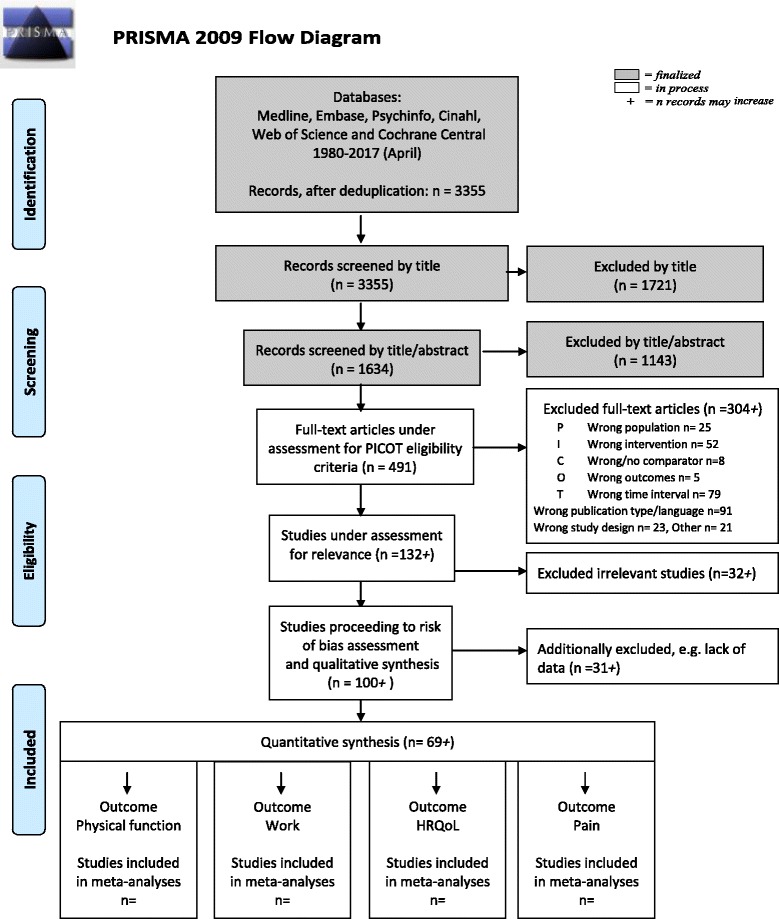
PRISMA flowchart illustrating the study selection process and planned structure of quantitative synthesis. From Moher D, Liberati A, Tetzlaff J, Altman DG, and the PRISMA group [19]

A PRISMA flow diagram [19] will be used to document the selection process, along with the reasons for exclusion (Fig. 1). We use EndNote reference software to organize, collate, and deduplicate search results. All records are saved in EndNote subfolders according to selection stage, for future reference. The online, systematic review production software, Covidence (www.covidence.org), will be used throughout the study selection procedure, archiving the full review process. Records are allocated to the reviewers by randomization and inter-rater agreement throughout the review process will be evaluated using appropriate analyses (kappa coefficients).

### Quality assessment

Articles deemed relevant from the full-text screening will be assessed for internal validity using the Quality In Prognostic Studies (QUIPS) tool [28]. QUIPS is designed to assess potential bias in prognostic factor studies which, preferably, use a prospective cohort design. The tool has been successfully used in several review projects with moderate/substantial inter-rater reliability. Risk of bias (RoB) will be evaluated within the six RoB domains: (1) study participation, (2) study attrition, (3) prognostic factor measurement, (4) outcome measurement, (5) study confounding, and (6) statistical analysis and reporting, and is rated individually as low, moderate, or high RoB. As recommended [22, 28], summary scores for overall study quality are avoided; thus, all assessed features will be presented in a complete RoB table and a RoB summary figure will be compiled for each outcome. RoB will be evaluated primarily at study level while comments on outcome-specific RoB will be noted for further detailing during data synthesis and sensitivity analyses. The RoB will also be incorporated in the judgment about the quality of evidence in the summary of findings and, if possible, in sensitivity analyses. All articles will be assessed independently by a senior epidemiologist (WG) and one of two reviewers (ET, PE) in accordance with the randomization scheme. Consensus on the final rating is reached through discussion. The process was piloted a priori on a small sample of studies for inter-rater agreement.

### Data synthesis

A digital coding protocol was created and pilot tested for data extraction on the first 10% of the included articles, before final revision. From each included study, data will be collected for six data domains: (1) participant and sample characteristics (including age, diagnosis, duration of pain, and inclusion and exclusion criteria), (2) characteristics of intervention (including type, professions and modalities involved, dose, duration, frequency, and setting), (3) investigated independent variables (= predictor/s) and how the information was collected, (4) outcome domains (dependent variables): pain, physical functioning, work ability, health-related quality of life, and how they were measured, (5) research design, length of follow-up, and percentage of loss to follow-up, and (6) statistical analyses and outcomes.

Data from the first third of included studies was extracted in duplicate, independently by two reviewers (WG, ET), and then compared for data accuracy and consensus on the coding procedure. The remaining studies will be coded independently for low inference data, whereas high inference data, on statistical outcomes and effect sizes, will be extracted jointly. If deemed relevant, clarification of reported data may be requested from investigators.

Currently, 69 articles are included in our study and the material encompasses in excess of 200 investigated predictive factors and their associations with our four predefined outcome domains. In these, preliminary data management indicated that work-related outcomes were investigated in the majority of articles while QoL was investigated in only a limited number: work (*n* = 47), physical function (*n* = 30), pain (*n* = 22), and QoL (*n* = 11). In order to competently sort and condense the material, our interdisciplinary review team will jointly perform a “consensus grouping process”; synonymous variables will be identified and labeled alike. Thereafter, all related predictive factors will be sorted into their pertinent domains, e.g., *personal* and *psychosocial predictors*, upon which coherent “predictor groups,” suitable for synthesis, will be identified, e.g., *emotional factors* and *cognitive behavioral factors.* Following this, predictors will be assembled into their related outcome domain, resulting in four outcome tables (pain, physical function, work, and QoL), which will constitute the basis for data synthesis.

#### Narrative synthesis

We will perform a narrative synthesis of all included studies in which the direction of the associations between predictors and outcomes will be presented as positive, negative, or absent in a tabular summary for each group of predictive factors. Data from both univariate and multivariate analysis models will be reported and included in the analyses. Depending on how data was presented in the original studies, results will, if necessary, be reversed to fit the chosen reporting direction of synthesis, i.e., for “good outcome” in every domain.

#### Quantitative synthesis

If the data proves to be appropriate for quantitative synthesis, meta-analyses will be performed. For pooling of predictor data pertaining to each outcome, at least two studies must provide data on the same predictor group, provided the judgment of study heterogeneity permits relevant summaries. Outcome data will be transformed to effect sizes and then to OR with corresponding 95% confidence intervals (95% CI), (if needed via standardized mean difference and standardized regression coefficients) according to what is suggested by Cooper (2009) [21] on research synthesis and meta-analysis. The strength of the relationships between identified predictors and corresponding outcomes will then be quantified using weighted pooled ORs in a random effects model for each outcome domain. A random effects model will be preferred for the statistical analysis since we expect substantial variability between studies related to both clinical and methodological diversity (heterogeneity). In the event of incomplete data for standardization, results will be reported in the narrative synthesis only. To avoid double counting [29], we plan to pool the data from studies that provide data from different predictive factors within the same predictor group. In this way, a study can only contribute with one predictive factor from the same predictor group. Statistical analyses will be conducted using the generic inverse-variance method in Review Manager software (RevMan, version 3. Copenhagen: The Nordic Cochrane Centre, The Cochrane Collaboration, 2014). When necessary, a web-based effect size calculator will be used for the purpose of computing and converting effect sizes [30].

### Sensitivity analyses

Analyses of heterogeneity will be assessed by sensitivity analyses to test the robustness of our results. Characteristics of studies that may be examined as potential causes of heterogeneity are pain diagnosis/duration, MDR-intervention profile/duration, RoB, and follow-up time. Subgroup analyses (e.g., diagnoses group) will be performed if possible. Funnel plots will be used to determine potential publication bias and heterogeneity of the included studies. Heterogeneity across studies will be assessed and quantified by the inconsistency index (I2). A summary table reporting any sensitivity analyses will be presented in the final report.

### Confidence in evidence

The strength of the emerging evidence will be evaluated using the Grading of Recommendations Assessment, Development, and Evaluation (GRADE) method [31]. The five GRADE domains consider confidence in estimates of treatment effect, i.e., risk of bias, imprecision, inconsistency, indirectness, and publication bias. GRADE can also readily be applied to bodies of evidence estimating longitudinal risks or prognosis of future events [32]. Applying GRADE domains to our included systematic reviews of prognostic studies will hence provide a useful approach to estimating confidence in the body of evidence included in our review. Any amendments to the stated procedure will be updated in PROSPERO and discussed in the final manuscript.

## Discussion

The present review project will use a rigorous methodology and provide an updated review of predictors of pain and physical functioning after MDR in patients with chronic pain*.* It examines published data with an explorative study approach to predictive factors rather than building on single predictor impact on the outcome, or on a predefined hypothesis as in traditional review study methodology—permitting an overview of factors central to MDR outcome. In previous reviews of predictors following MDR in patients with chronic musculoskeletal pain problems, it has been judged impossible to conduct meta-analyses [11–13] due to lack of power and heterogeneity of studies. Also, by investigating one predictor domain at a time, and their specific contributions in relation to other possible factors, the big picture is easily overlooked. A thorough overview of studied factors that might predict clinically important outcomes is therefore needed.

A strength of this review project is that it aims for the meta-analysis of prospective cohort or randomized controlled studies by performing an extensive search of multiple databases, journals, references, and citations, combined with consulting experts and independent reviewers throughout the study process. A wide array of investigated variables will be dealt with using the same systematic conduct and analyses—which will provide a consistent evidence synthesis. However, our initial review procedure has resulted in large volumes of relevant published data, and we, therefore, believe it is reasonable to outline more than one review article relating to our primarily defined outcomes—the first paper will focus solely on physical functioning.

Regarding potential limitations, our review omitted all studies not published in English as well as gray literature, which may contribute to limitations related to publication bias. Also, the broad approach to the research question may present challenges for data synthesis. For example, the choice to pool different predictors assessing the same construct into one predictive factor per study and to pool different outcomes that assess the same construct into one outcome per study could lead to a lack in precision of the estimates, but we believe it is crucial to avoid any double reporting of data. Hence, our interdisciplinary research group will continuously provide necessary expertise and input into decisions and delimitations, e.g., when categorizing investigated factors into logical and theoretically coherent predictor groups and in defining homogenous outcome measures for the pooling of estimates.

Our intention is to add and update existing data on predictive factors related to outcome of MDR treatment in chronic pain, thereby strengthening the evidence base for improving healthcare and public health policy. This updated body of evidence will hopefully provide support for clinical centers delivering MDR and enable them to further optimize rehabilitation in this major patient group with chronic pain.

As a result of detailed scrutiny of primary studies, our systematic review may also identify the strengths and limitations of research in this specific field and provide recommendations for future investigations. The simple reiteration of prognostic factor research has been recommended against due to its limitations in providing clinical guidance, instead the recommendation for taking prognosis research one step further is to pursue the development of clinical prediction tools. We believe that this study will inspire the building of prediction tools and provide a framework for their development. The authors of this protocol and our research network are all linked to the Swedish Quality Registry for Pain Rehabilitation and have access to rich, longitudinal clinical registry data that will enable further prognostic research to spring from real world practice.

In sum, findings will be disseminated widely including publication in peer-reviewed journals and through conferences. Although the risk of bias and heterogeneity may limit the overall strength of evidence, we believe our results will provide a better understanding of important predictors in patients with chronic pain following MDR. Our results will, we trust, help a wide audience including health care practitioners and policymakers. They will provide a solid basis of evidence that will be indispensable in our continued endeavor to develop and validate a clinical prediction tool relevant for this major patient group.

## Additional files

Additional file 1: PRISMA –P checklist. (DOCX 30 kb)

Additional file 2: Medline Search strategy. (DOCX 22 kb)

## Abbreviations

GRADE: Grading of Recommendations, Assessment, Development and Evaluation
ICF: International Classification of Functioning, Disability, and Health
IMMPACT: Initiative on Methods, Measurement, and Pain Assessment in Clinical Trials
MDR: Multidisciplinary rehabilitation
MeSH: Medical Subject Heading-indexation
PICO: Population, intervention, comparator, outcome
PRISMA-P: Preferred Reporting Items for Systematic Review and Meta-Analysis protocols
PROSPERO: International Prospective Register of Systematic Reviews
QUIPS: Quality in Prognostic Studies tool
RCT: Randomized controlled trial
RoB: Risk of bias
YLD: Years lived with disability

## References

[1] Leadley RM, Armstrong N, Lee YC, Allen A, Kleijnen J (2012). Chronic diseases in the European Union: the prevalence and health cost implications of chronic pain. J Pain Palliat Care Pharmacother.

[2] Turk DC, Dworkin RH, Revicki D, Harding G, Burke LB, Cella D (2008). Identifying important outcome domains for chronic pain clinical trials: an IMMPACT survey of people with pain. Pain.

[3] Global Burden of Disease Study C (2015). Global, regional, and national incidence, prevalence, and years lived with disability for 301 acute and chronic diseases and injuries in 188 countries, 1990-2013: a systematic analysis for the Global Burden of Disease Study 2013. Lancet.

[4] Ahlberg M, Axelsson S, Eckerlund I, Gerdle B, Stålnacke B-M, Söderlund A (2010). Rehabilitering vid långvarig smärta: en systematisk litteraturöversikt. SBU—Statens beredning för medicinisk utvärdering.

[5] Gatchel RJ, McGeary DD, McGeary CA, Lippe B (2014). Interdisciplinary chronic pain management: past, present, and future. Am Psychol.

[6] Craig P, Dieppe P, Macintyre S, Michie S, Nazareth I, Petticrew M (2008). Developing and evaluating complex interventions: the new Medical Research Council guidance. BMJ.

[7] Turk DC, Dworkin Rh, Allen RR, Bellamy N, Brandenburg N, Carr DB, Cleeland C, et al. Core outcome domains for chronic pain clinical trials: IMMPACT recommendations. 2003(0304–3959 (Print)). 10.1016/j.pain.2003.08.001 14659516

[8] Taylor AM, Phillips K, Patel KV, Turk DC, Dworkin RH, Beaton D, Clauw DJ, et al. Assessment of physical function and participation in chronic pain clinical trials: IMMPACT/OMERACT recommendations. (1872–6623 (Electronic)). 10.1097/j.pain.0000000000000577 PMC7453823 27058676

[9] WHO (2001). International Classification of Functioning, Disability and Health (ICF).

[10] Scascighini L, Toma V, Dober-Spielmann S, Sprott H (2008). Multidisciplinary treatment for chronic pain: a systematic review of interventions and outcomes. Rheumatology.

[11] de Rooij A, Roorda LD, Otten RH, van der Leeden M, Dekker J, Steultjens MP (2013). Predictors of multidisciplinary treatment outcome in fibromyalgia: a systematic review. Disabil Rehabil.

[12] van der Hulst M, Vollenbroek-Hutten MM, Ijzerman MJ (2005). A systematic review of sociodemographic, physical, and psychological predictors of multidisciplinary rehabilitation—or, back school treatment outcome in patients with chronic low back pain. Spine (Phila Pa 1976).

[13] Wessels T, van Tulder M, Sigl T, Ewert T, Limm H, Stucki G (2006). What predicts outcome in non-operative treatments of chronic low back pain? A systematic review. Eur Spine J.

[14] Taylor AM, Phillips K, Taylor JO, Singh JA, Conaghan PG, Choy EH (2015). Is chronic pain a disease in its own right? Discussions from a Pre-OMERACT 2014 workshop on chronic pain. J Rheumatol.

[15] Artus M, Campbell P, Mallen CD, Dunn KM, van Der Windt DAW (2017). Generic prognostic factors for musculoskeletal pain in primary care: a systematic review. BMJ Open.

[16] Valentin GH, Pilegaard MS, Vaegter HB, Rosendal M, Ørtenblad L, Væggemose U (2016). Prognostic factors for disability and sick leave in patients with subacute non-malignant pain: a systematic review of cohort studies. BMJ Open.

[17] Laisné F, Lecomte C, Corbière M (2012). Biopsychosocial predictors of prognosis in musculoskeletal disorders: a systematic review of the literature (corrected and republished). Disabil Rehabil.

[18] Moher D, Shamseer L, Clarke M, Ghersi D, Liberati A, Petticrew M (2015). Preferred reporting items for systematic review and meta-analysis protocols (PRISMA-P) 2015 statement. Syst Rev.

[19] Moher D, Liberati A, Tetzlaff J, Altman DG, The PRISMA Group (2009). Preferred reporting items for systematic reviews and meta-analyses: the PRISMA statement. BMJ.

[20] Turk DC, Dworkin RH, Allen RR, Bellamy N, Brandenburg N, Carr DB (2003). Core outcome domains for chronic pain clinical trials: IMMPACT recommendations. Pain.

[21] Cooper H, Hedges LV, Valentine JC. The handbook of research synthesis and meta-analysis. 2nd ed. New York: Russell Sage Foundation; 2009.

[22] Higgins JPT GSe. Cochrane Handbook for Systematic Reviews of Interventions. Version 5.1.0 [updated March 2011]. The Cochrane Collaboration, 2011. Available from http://handbook.cochrane.org. 2011.

[23] Kamper SJ, Apeldoorn AT, Chiarotto A, Smeets RJEM, Ostelo RWJG, Guzman J (2015). Multidisciplinary biopsychosocial rehabilitation for chronic low back pain: Cochrane systematic review and meta-analysis. BMJ.

[24] van Geen JW, Edelaar MJ, Janssen M, van Eijk JTM, van Eijk JT. The long-term effect of multidisciplinary back training: a systematic review. (1528–1159 (Electronic)). 10.1097/01.brs.0000251745.00674.08 17224822

[25] Cooper HM. Research synthesis and meta-analysis: a step-by-step approach. 4th ed. ed. Cooper HM, editor. Los Angeles, [Calif.]: London: SAGE; 2010.

[26] Jenuwine ES, Floyd JA (2004). Comparison of medical subject headings and text-word searches in MEDLINE to retrieve studies on sleep in healthy individuals. J Med Libr Assoc.

[27] Geersing G-J, Bouwmeester W, Zuithoff P, Spijker R, Leeflang M, Moons K (2012). Search filters for finding prognostic and diagnostic prediction studies in Medline to enhance systematic reviews (finding prediction research using search filters). PLoS One.

[28] Hayden JA, van der Windt DA, Cartwright JL, Cote P, Bombardier C (2013). Assessing bias in studies of prognostic factors. Ann Intern Med.

[29] Senn SJ (2009). Overstating the evidence—double counting in meta-analysis and related problems. BMC Med Res Methodol.

[30] Wilson DB. Practical meta-analysis effect size calculator. Campbell Collaboration.org; 2015.

[31] Atkins D, Best D, Briss PA, Eccles M, Falck-Ytter Y, Flottorp S, Guyatt GH, et al. Grading quality of evidence and strength of recommendations. (1756–1833 (Electronic)). 10.1136/bmj.328.7454.1490 PMC428525 15205295

[32] Huguet A, Hayden JA, Stinson J, McGrath PJ, Chambers CT, Tougas ME (2013). Judging the quality of evidence in reviews of prognostic factor research: adapting the GRADE framework. Syst Rev.

