# Supplementary material for: Predictors of multidisciplinary rehabilitation outcomes in patients with chronic musculoskeletal pain: protocol for a systematic review and meta-analysis
Source: Syst Rev. 2017 Oct 11;6:199. doi: 10.1186/s13643-017-0598-0 (PMC5637325; doi:10.1186/s13643-017-0598-0)
Supplement: Supplementary file 2 — Medline Search strategy. (DOCX 22 kb) [file 13643_2017_598_MOESM2_ESM.docx]

**ADDITIONAL FILE 2**

**Medline Search Strategy**
